# Nanoparticles: a new frontier in neurodegenerative disease therapy

**DOI:** 10.3389/fmedt.2025.1654003

**Published:** 2025-11-20

**Authors:** Vipin Kumar, Shivani Sharma, Anchal Singh

**Affiliations:** Department of Biochemistry, Institute of Science, Banaras Hindu University, Varanasi, India

**Keywords:** neurodegenerative diseases, blood-brain barrier, CNS therapeutics, nerve growth factor, neural stem cells, nanocrystals

## Abstract

Nanoparticle-based drug delivery systems, such as liposomes, polymeric micelles, dendrimers, and other nanosized carriers, have emerged as promising strategies to improve the targeted delivery of therapeutic agents to the brain. These nanoparticles can be engineered to encapsulate drugs, facilitating their passage across the BBB, enabling localized treatment of the regions affected by neurodegeneration. Nanoparticles are characterized by their small size, large surface area, and possibility of functionalization, which allows them to be useful in many areas, including improved bioavailability, decreased systemic side effects, and improved therapeutic efficacy. Additionally, nanoparticles may also be surface-modified with appropriate ligands like antibodies, peptides, or small molecules, which exhibit specific interactions with receptors or cellular targets associated with the disease process. Such targeting has the potential to make targeted drug delivery possible, allowing therapeutic factors that can damage the healthy part of the brain to be delivered only to the diseased region. Furthermore, probable treatments for neurodegenerative diseases are also reviewed with the potential for complexation of different therapeutic agents, including small molecules, proteins, RNA, lipid nanoparticles and gene therapies with nanoparticle-based systems.

## 1 Introduction

**Figure 1:**
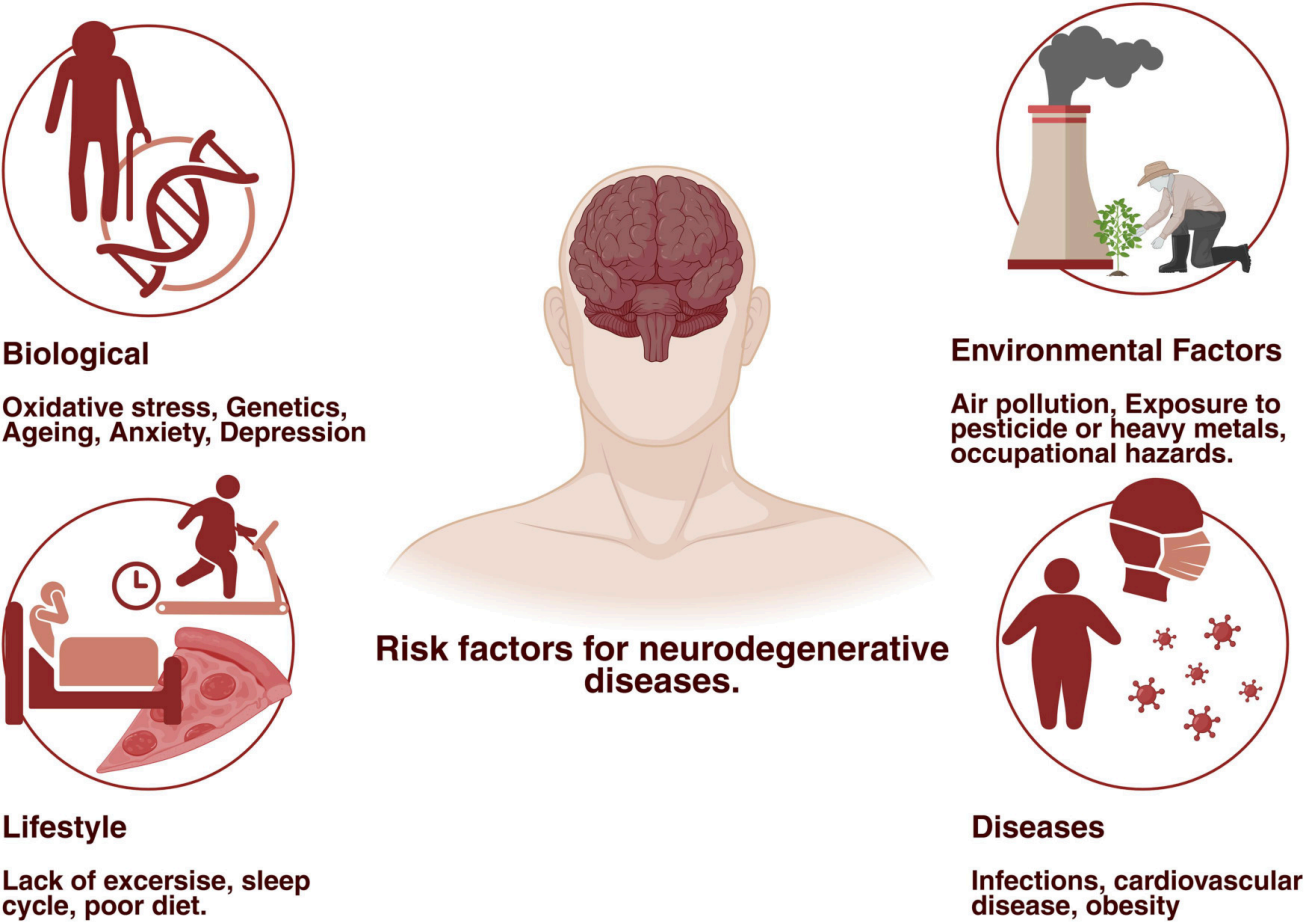
Factors contributing to neurodegenerative diseases. The diagram illustrates the major risk factors which are associated with the development and progression of neurodegenerative diseases. These includes: biological factors- such as ageing, genetics, oxidative stress, anxiety, depression. Environmental factors- like air pollution, pesticides, heavy metals, and occupational hazards. Lifestyle- habits like lack of physical activity, irregular sleep cycle, poor diet. Diseases- including obesity, cardiovascular disorders, and chronic infections. Together these factors contribute to compromising brain health deterioration and accelerate the neurodegenerative processes. Created in BioRender. Sharma, S. (2025) https://BioRender.com/vjbce88

Neurodegenerative diseases (NDs) are the prominent source of disability and cause approximately 12% of all deaths at the global level. Central nervous system (brain, retina, and spinal cord) neurodegenerative illnesses impact people of all ages (1) and can include congenital leukodystrophies that affect the white matter in children and become more common as people age, like age-related macular degeneration (AMD), Parkinson's disease (PD), and Alzheimer's disease (AD). Degenerative mental diseases, like schizophrenia, can cause cerebral grey matter loss and symptoms of accelerated aging in their sufferers (2). Other NDs, like Huntington's disease (HD), amyotrophic lateral sclerosis (ALS), frontotemporal dementia (FTD), corticobasal syndrome (CBS), multiple system atrophy (MSA), and progressive supranuclear palsy (PSP), are less common, while AD, AMD, and PD are more common (3) (Figure 1).

Although some disorders, such as Huntington's disease and ALS, frequently manifest earlier, the majority of these conditions are closely linked to age and are far more likely to occur in those over 65 years of age (4). The condition is more worrisome as the number of individuals over 65 is expected to at least double over the next 30 years, according to World Health Organization (WHO) projections. This implies that the number of persons suffering from NDs will likewise increase at a comparable pace (Figure 1). Even though the pathophysiology of these conditions is well understood, it has not been adequately translated into effective treatments to stop or reverse the course of the disease (5).

One of the key challenges in developing effective therapies against NDs is the blood-brain barrier (BBB) which is very selective hence, hinders the delivery of therapeutic agents to the brain (6). This hurdle markedly reduces the efficiency of classical drug delivery systems, and thus poses a serious challenge for the treatment of NDs. Novel strategies have been established based on advances in nanotechnology to cross the BBB (7). BBB prevents nearly 99% of small molecules, as well as a significant proportion of biotherapeutics, from reaching their intended targets within the brain (8). Although the brain constitutes only approximately 2% of the body weight, it is highly vascularized, receiving nearly 20% of the cardiac output. While systemic administration of therapeutics has been commonly used to treat CNS (Central Nervous System) disorders, this approach faces notable hurdles that diminish the success of many neural treatments (9). These challenges highlight the requirement for an improved delivery system that can overcome the BBB and facilitate targeted delivery to specific pathological brain regions or cell types. A specific non-viral delivery system that achieves optimized CNS penetration and targeted delivery to pathological brain regions is the missing link in CNS therapeutic development (10). Undoubtedly, the unavailability of such systems has therefore fundamentally hampered the advancement of efficacious CNS therapeutics (11).

Recent breakthroughs in nanotechnology have provided novel approaches for overcoming the BBB. NPs have many benefits in drug delivery, including flexibility, ease of biodegradability, and the ability to penetrate the BBB. Size, surface charge, and flexibility are important determinants for cellular internalization of NPs, with lipid-based NPs being more readily internalized than rigid silica or metal-based NPs (12, 13). Furthermore, NPs may be designed to allow active or passive transport and targeted drug release, such as endocytosis or diffusion through cell membrane spaces. Stimuli-responsive NPs, which are sensitive to pH, temperature, and reactive oxygen species (ROS), are considered better for calculated drug release. NPs can be customized for administration in a variety of diseases including NDs, as many of these diseases like AD, PD, and Friedreich's ataxia are due to abnormal ROS production. Additionally, NPs may help address challenges with NDs' treatment, including poor bioavailability and toxicity of conventional drugs (14).

The complexity of the physiological and pathophysiological barriers encountered during the systemic delivery of NPs to the brain complicates the development of successful NP-based therapeutics (15). Initially, NPs must pass the physiological barriers in the systemic circulation post-administration that may eliminate nanoparticles from the bloodstream (16). After this, NPs must cross the BBB, a highly selective barrier formed by endothelial cells in the brain blood vessels that limits the diffusion of most substances, including therapeutic agents. Besides the BBB, NPs can be engineered to overcome the complex signalling, cellular, and tissue-specific barriers within the brain that shape their fate and efficacy to reach the target site. Designing efficient NP systems should address such challenges encountered at each and every step of the delivery process (17, 18). A complete understanding of the brain anatomy and physiology along with disease pathological processes, as well as the specific molecular and cellular mechanisms regulating NPs behaviour in the brain, is needed to translate NPs into clinically useful technologies. In addition, specific delivery of NPs to the regions of brain and/or brain cells responsible for specific diseases is essential for its therapeutics. For example, AD, PD, and brain tumors will require different methodologies for targeting of various brain cells (neurons, glial cells, tumor cells, etc.) involved in each pathology. Thus, the advancement of precision delivery systems for CNS ailments necessitates an interdisciplinary trajectory merging prowess in nanotechnology, molecular biology, and neuroscience (19).

The review intends to give a broad and impartial presentation of the present status of nanoparticle-mediated drug delivery approaches for CNS disorders. An overview of recent developments in the field of nanoparticle-mediated CNS drug delivery and a brief discussion of significant hurdles to drug delivery to the CNS and the progress made so far in overcoming such hurdles for traditional CNS therapies have been attempted here. It also aims to lay out the key areas of research needed to accelerate the development of new and efficacious precision nano-delivery systems that can transform the treatment of CNS diseases in the near future.

## 2 Size and properties of nanoparticles

Nanoparticles (NPs), typically ranging from 1 to 100 nm, are synthesized from various materials like metals, polymers, and carbon compounds (20). They can be classified as organic (lipid-based, e.g., micelles, liposomes, nanoemulsions), polymeric (e.g., polymer micelles, dendrimers), or inorganic (e.g., carbon nanotubes, gold and iron oxide NPs) (Table 1) (21). Due to their unique properties, such as high surface-to-mass ratio, stability, and ability to interact with both hydrophilic and hydrophobic environments, NPs show great promise in treating NDs, as their ability to mimic cell membrane structures and encapsulate diverse pharmaceuticals, along with their potential for functionalization, enhances their usefulness in drug delivery and bio-sensing (22). NP's have the ability to improve pharmacokinetics, and reduce required drug dosages, to minimize systemic side effects, which makes them particularly lucrative as therapeutics (23). Nanoemulsions, for instance, are nanoscale emulsions that enable easier administration and absorption, while nanocrystals offer solid carriers for drug delivery (24, 25). Preclinical studies on gold nanocrystals have shown promise in treating neurodegenerative conditions by improving neurorepair and neuronal resilience, with positive results in clinical trials (26) (Table 1). However, NPs usage has certain challenges, such as including ROS-induced toxicity, abnormal internalization into tissues, and aggregation. Exposure to NPs through inhalation or systemic administration can cause NPs accumulation in organs such as the lungs, liver, or kidneys with inflammation or neurotoxicity (27). Future developments should aim to improve NPs clearance, by: (i) refining targeting mechanisms (e.g., receptor-mediated transcytosis), and (ii) imparting a “stealth-coating” with polymers or signalling proteins to extend the circulation time of NPs.

**Table 1:** Nanoparticle used for the treatment of neurodegenerative diseases.

Nanoparticle type	Active reagent of nano drugs	Application of nano drugs	Clinical status	Disease	Ref.
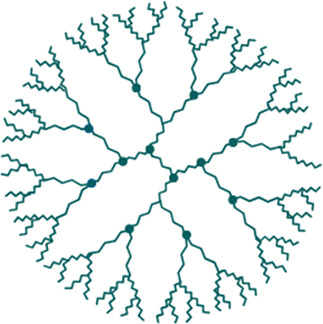 Dendrimers	Memantine, Pioglitazone	Enhanced drug solubility, BBB penetration, and anti-inflammatory effects	Preclinical Studies	Alzheimer's Disease (AD)	(43, 118, 119)
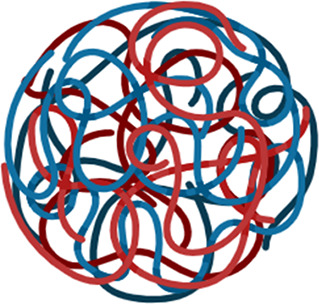 Polymeric Nanoparticles	Rivastigmine, Galantamine	Sustained drug release, improved bioavailability, and targeted delivery to the brain	Preclinical and Clinical Trials	Alzheimer's Disease (AD)	(43, 174)
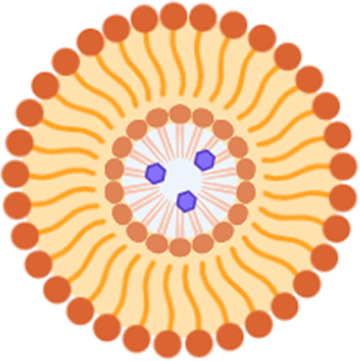 Nano emulsions	Cannabidiol (CBD)	Improved bioavailability and anti-inflammatory effects	Preclinical Studies	Alzheimer's Disease (AD)	(24, 25)
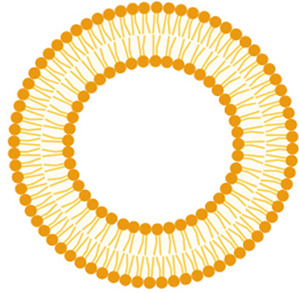 Liposomes	Curcumin, Donepezil	Enhanced blood-brain barrier (BBB) penetration, anti-inflammatory, and antioxidant effects	Preclinical and Clinical Trials	Alzheimer's Disease (AD)	(45)
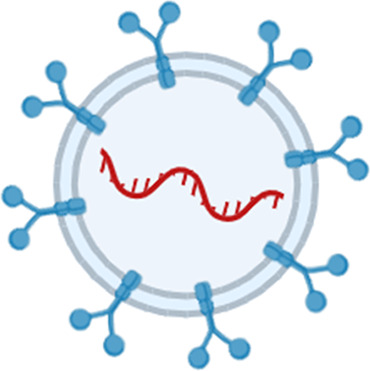 Exosomes	miRNA, Growth Factors	Natural carriers for drug delivery, promote neurogenesis and reduce inflammation	Preclinical Studies	Parkinson's Disease (PD)	(83)
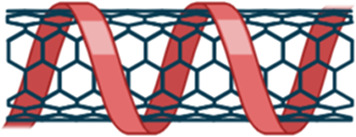 Carbon Nanotubes	Levodopa	Improved drug delivery and sustained release of dopamine precursors	Experimental Stage	Parkinson's Disease (PD)	(21)
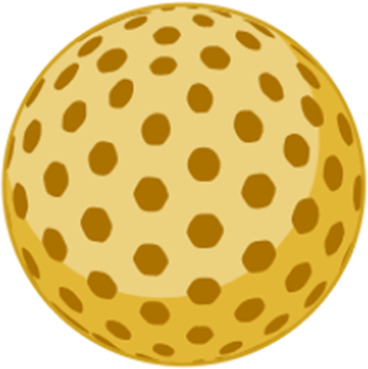 Mesoporous Silica Nanoparticles	Nicotinamide, Coenzyme Q10	Controlled drug release, antioxidant, and mitochondrial support	Preclinical Studies	Parkinson's Disease (PD)	(181), (https://clinicaltrials.gov/study/NCT03815916?term=NCT03815916&rank=1)
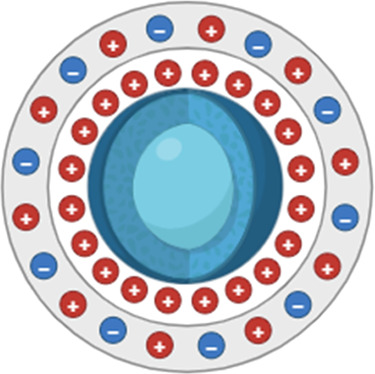 Magnetic Nanoparticles	Iron Oxide, Antioxidants	Targeted delivery, imaging, and reduction of oxidative stress	Preclinical Studies	Alzheimer's Disease (AD)	(74, 75)
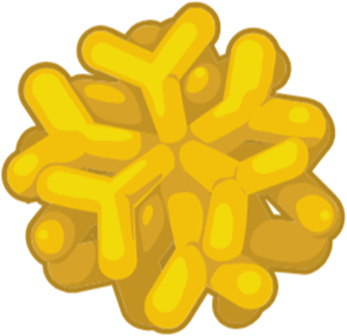 Gold Nanoparticles	Antioxidants, siRNA	Targeted delivery, reduced oxidative stress, and gene silencing	Preclinical Studies	Parkinson's Disease (PD)	(75), (https://clinicaltrials.gov/study/NCT03815916?term=NCT03815916&rank=1)

The table summarises the types of nanoparticles their active reagents, applications in terms of targeting delivery in BBB, clinical status, and the disease in which it plays the role.

## 3 Current therapies for NDs: Challenges and Roadblocks

### 3.1 Amyotrophic Lateral Sclerosis

ALS, commonly referred to as motor neuron disease (MND) or Lou Gehrig's disease, is characterized by its rapid progression and neurodegenerative nature. Degeneration of the lower motor neurons in the brainstem and spinal cord and the upper motor neurons in the motor cortex results in gradual denervation of voluntary muscles, leading to disruption of neural networks through a complex set of mechanisms eventually culminating in motor and non-motor symptoms (28). Globally 10%–21% cases of ALS are caused by monogenic alleles, however, in the majority of cases, a combination of various genetic and environmental risk factors is involved. Approximately 5%–10% of ALS patients have a family history of the diseases that follows the Mendelian autosomal dominant pattern of inheritance and more than that 60%–70% of familial ALS (fALS) is caused by genetic defects. Even 90%–95% cases of sporadic ALS (sALS) are caused due to genetic factors, in patients who do not have any family history. It has been determined that over 30 genes either cause ALS or enhance the risk drastically; up to 70% of fALS cases are majorly attributed to four genes: C9orf72, SOD1, TARDBP, and FUS (29), while other ALS-causing genes and their roles are enlisted in Table 2.

**Table 2:** Pathophysiological mechanism of amyotrophic lateral sclerosis.

Genes	Disease mechanism	Phenotypic features	Pathological effects	Ref.
TDP-43, SOD1, FUS, ubiquilin	Protein aggregation	Muscle weakness, atrophy cytoplasmic inclusions in motor neurons	Misfolded proteins accumulate and form insoluble inclusions in neurons and glia	(30, 32, 51)
SOD1, ROS, lipid peroxidation	Oxidative stress	Fatigue, progressive motor decline and muscle wasting	Excess reactive oxygen species damage proteins, lipids, and DNA	(30, 32)
SETX, FUS, ATM	DNA damage/impaired repair	Juvenile ALS, cognitive symptoms and motor neuron loss	Failure in DNA repair increases neuronal vulnerability and accelerates degeneration	(36)
Glutamate, AMPA/NMDA receptors, EAAT2	Excitotoxicity	Spasticity, hyperreflexia cramps and fasciculations	Excess glutamate overstimulates neurons, causing calcium overload and death	(31)
CHCHD10, VCP, PGC-1α	Mitochondrial dysfunction	Early-onset fatigue, energy failure and bulbar symptoms	Impaired ATP production and increased ROS generation cause neuron death	(39)
TDP-43, FUS, C9ORF72, hnRNPA1	Dysregulated RNA metabolism	Motor neuron degeneration, progressive weakness and dysarthria	Impaired RNA splicing, transport, and translation due to mutations in RNA-binding proteins	(36, 39, 40)
TNF-α, IL-1β, NF-*κ*B, microglia, astrocytes	Neuroinflammation	Rapid progression, worsening motor decline, gliosis in CNS	Activated glial release inflammatory mediators that damage motor neurons	(35, 34)
C9ORF72, TDP-43, nuclear pore proteins	Nucleocytoplasmic transport defects	TDP-43 mislocalization, behavioral variant ALS (ALS-FTD overlap); bulbar or limb onset	Impaired shuttling of proteins and RNAs between nucleus and cytoplasm	(32)

Excess reactive oxygen species (ROS), reactive nitrogen species, or compromised antioxidant defense systems can all lead to oxidative stress. Many of the oxidative stress biomarker profiles are altered in ALS bio-samples and animal models, where it has been observed that oxidative stress promotes the aggregation of acetylated TDP-43 which hinders RNA binding and encourages the accumulation of insoluble, hyperphosphorylated TDP-43 species. The TDP-43 aggregate sequesters microRNAs and proteins such as mitochondrial proteins encoded by the nuclear genome resulting in dysregulation of mitochondria and amplified oxidative stress. In cellular models of ALS the RNA processing gets severely impaired upon oxidative stress, in turn causing the mis-localization of TDP-43 and FUS (30).

Another major factor contributing to the pathophysiological mechanisms in ALS is excitotoxicity, triggered by excessive postsynaptic glutamate receptor stimulation. Prolonged synaptic glutamate elevation causes excessive neuronal firing, which raises intracellular calcium levels and has subsequent neurotoxic effects. Continuous pathogenic alterations such as mitochondrial calcium buildup and ER stress are brought about by excitotoxicity. The glutamate reuptake transporter GLT1–EAAT2 is reduced in the CNS of ALS patients and also in animal models. Mutations in ALS-related genes interfere with mitochondrial functioning that leads to problems in energy production, increased oxidative stress, and abnormal mitochondrial axonal transport (31). Numerous aberrant ALS associated proteins can directly interact with mitochondria, compromising their functioning, for instance mutant SOD1 protein accumulates inside mitochondria and impairs energy-generating complexes. Similarly, proteins from C9orf72 mutations interfere with mitochondrial components and disrupt energy balance. TDP-43 also influences mitochondria by regulating mitochondrial RNA, but upon mutation it impairs mitochondrial function, leading to an increase in oxidative stress (32). In ALS, the proteostasis system loses control over processes like protein synthesis, folding, and degradation resulting in the buildup of damaged or misfolded proteins, like SOD1 and TDP-43 may form aggregates (33). C9ORF72, sequestosome 1/P62, optineurin, and ubiquilin 2 initiate autophagy; alsin, FIG4, VCP, and CHMP2B control autophagosome maturation; and TBK1 loss of function inhibits substrate transport to autophagosomes (Table 2).

Inflammation within the nervous system, particularly involving glial cells, has been one of the causative factors for the development of ALS. Astrocytes from ALS patients release inflammatory molecules instead of providing energy support, which is often responsible for microglia shifting to an inflammatory state incurring neuronal damage (34). Two important regulators of this inflammatory response are the NLRP3 inflammasome and the NF-κB signaling pathway, and blocking these pathways may provide therapeutic benefits (35). NF-κB signaling is implicated as a key regulator of microglial activation in ALS, as in mutant SOD1 mice, this signalling was triggered in glia as the disease progressed (Table 2). The deletion of NF-κB signalling in microglia rescued motor neurons from microglia-mediated death and prolonged neuronal survival in ALS mice by preventing pro-inflammatory microglial activation (34).

Neurons and other post-mitotic cells are particularly vulnerable to DNA damage, which results in cell death if left untreated. The concentration of deoxyguanosine (OdG) is increased in CNS tissue and biofluid samples in ALS patients having DNA damage. According to recent reports, ALS is associated with increased apurinic/apyrimidinic DNA sites and activation of DNA damage response (DDR). It has also been demonstrated that in spinal cord tissue from sALS patients, loss of nuclear TDP-43 correlates with strand breaks and DDR response, as some *in vitro* DNA damage cell models revealed that TDP-43 is involved in non-homologous end-joining-mediated repair. Dysregulation of DNA repair has been linked to mutations in NEK1 and FUS. Numerous lines of evidence have already shown the lack of functioning of DDR or DNA repair genes is linked to ALS development (36). Motor neurons are dependent on retrograde and anterograde transport of cargos to maintain axonal integrity, and any defect in axonal transport can lead to ALS as seen in ALS models and humans alike (37). ALS models often exhibit transport failure, causing the accumulation of organelles in axons and defects linked to mutations in transport-related genes like KIF5A and ANXA11 (38).

ALS progression has been attributed to the disruption of RNA processing in several instances. The proteins TDP-43 and FUS are involved in managing RNA, and influencing thousands of genes, and their dysfunctioning severely impacts RNA splicing, stability, and export. Mutations in C9orf72 result in aberrant RNA repeats that trap proteins such as TDP-43, causing RNA metabolism to be disrupted and toxic dipeptide repeat proteins (DPRs) to be produced. Anti-sense nucleotides (ASOs) have demonstrated promise in lowering RNA foci and their deleterious effects and ASOs are under clinical trials for ALS treatment (39, 40).

### 3.2 Alzheimer's disease (AD)

AD is a progressive ND characterized by neurofibrillary tangles produced by tau proteins as well as a buildup of amyloid plaques (41). As the world's population is aging, the prevalence of AD will increase dramatically such that by 2050 the number of people with AD in the U.S. alone may reach 14 million, or triple the current disease burden (42). Today, the available treatments are mostly symptomatic rather than treating the underlying disease. There are several drugs, including acetylcholinesterase inhibitors (donepezil, rivastigmine, tacrine) and memantine (an NMDA receptor antagonist), and galantamine that are FDA approved to manage AD symptoms (43). These medications aim to enhance cholinergic function in the brain, which is often compromised in AD patients. Notwithstanding their widespread use, these treatments have been shown to provide only modest benefits, mainly in terms of cognitive and functional improvement, and do not stop disease progression. Antipsychotics like olanzapine, quetiapine, etc., are much less effective and can cause oppressive side effects when used off-label for behavioural symptoms (44).

However, recent investigations of alternative therapies for AD have sparked interest in compounds with disease-modifying capability, one such molecule, a natural polyphenol, is curcumin, which has anti-inflammatory and antioxidant properties (45). Animal model studies of AD indicate that curcumin could reduce amyloid plaque deposition and brain inflammation, although clinical trials in humans are quite early (Table 1). An alternative strategy is based on targeting tumor necrosis factor alpha (TNF-α), a pro-inflammatory cytokine that has increased levels in AD. The work identified the TNF-α receptor as a promising AD target, and etanercept, a recombinant DNA-based anti-TNF-α drug, reduces AD development, supporting anti-inflammatory strategies in AD (46). Gene therapy has also been tried in AD where genetic modifications of Nerve Growth Factor (NGF) is performed, which is a key element required for the survival, and functioning of the cholinergic neurons. It has been shown in clinical trials that the survival of these neurons can be increased with NGF therapy, leading to reduced cognitive decline in AD patients. Nonetheless, advances are necessary with regard to the delivery, since intracranially injected NGF has thus far not translated into meaningful changes in morbidity and mortality (47).

Another promising AD therapeutic approach is employing monoclonal antibodies (mAbs) that target the amyloid plaques. Bapineuzumab (AAB-001) is a humanized mAb developed by Elan and Wyeth that binds to amyloid plaques in the CNS and promotes their clearance. Clinical trials of Bapineuzumab are mixed, with some Bapineuzumab studies suggesting mild cognition improvements and others suggesting little efficacy in slowing disease progression. Conversely, other anti-amyloid mAbs (e.g., aducanumab) have demonstrated more favourable outcomes in recent trials, culminating in an FDA endorsement for treatment of early AD stages (dementia or mild cognitive impairment) (48). Despite significant research and development efforts to identify effective therapies, no drug currently exists that can slow or stop progression of AD. This means that the most effective treatment plan typically includes both symptomatic management and disease-modifying therapies. Novel approaches, including immunotherapy, gene therapy, and anti-inflammatory agents, have been explored, but further studies are necessary to establish their clinical efficacy and safety in patients with AD (49).

#### 3.2.1 Parkinson's disease (PD)

PD is a CNS movement disorder that primarily occurs in people over 60 years, with an estimated 1% prevalence in the population belonging to this age group. In PD there is slow destruction of dopaminergic neurons in the substantia nigra, resulting in reduced dopamine levels in the brain (50). Neuronal death is due to the aggregation of ubiquitinated α-synuclein, a well-known pathophysiological marker of PD. The presence of a group of symptoms together, such as tremors, bradykinesia and (in more advanced cases) akinesia is referred to clinically as parkinsonism (51). At present, PD therapeutic options are mainly symptomatic, for e.g., dopamine replacement and agonists, monoamine oxidase (MAO) inhibitors, catechol-O-methyl transferase (COMT) inhibitors, anti-cholinergic drugs, and amantadine are standard symptomatic treatments for motor symptoms, however, none of these therapies either cures or prevents consistent neurodegeneration. Consequently, this necessitates therapies that effectively relieve symptoms while also offering neuroprotection and deceleration of the degenerative processes that characterize PD.

Neurotrophic factors like glial cell-derived neurotrophic factor (GDNF) and brain-derived neurotrophic factor (BDNF) have emerged as possible agents to promote dopaminergic neuron survival and function in preclinical PD models (52). Moreover, the development of new RNA-based therapeutic strategies, especially RNA interference (RNAi) therapies, also appears as a possible therapeutic alternative to reduce expression of pathogenic α-synuclein (53). RNAi has shown potential in animal models of PD to selectively target the mutated α-synuclein gene, thus preventing neurotoxicity. However, problems in entering into the CNS prevented RNAi-based antidotes from moving to the clinical stage. Hence, ensuring that RNAi constructs can get through the blood-brain barrier without breaking down and can accurately hit their target in the brain is of prime importance for the PD patients. In addition, alternative ways of administration, for example, intranasal delivery, have been examined as a non-invasive approach for improving CNS drug delivery. Intranasal administration avoids the BBB by using the olfactory and (to a lesser extent) trigeminal nerves, offering a direct corridor to the brain, thus improving the chances of successful therapeutic outcomes (54). While such novel remedies hold promise, more research is needed to fine-tune these delivery systems and examine their long-term safety and efficiency in clinical applications. Advanced drug delivery strategies and developing effective treatments, which can not only alleviate the symptoms of PD but also attack its root causes, are the need of the time.

### 3.3 Huntington's disease (HD)

HD is caused by a mutation in the Huntingtin protein, which results in the aggregation of this protein in the neurons of HD patients (55). Upon disease progression, patients develop abnormal movements, known as chorea. Eventually, Huntingtin aggregation interrupts the function of neurons, culminating into death of HD patients. HD is an autosomal dominant genetic disease, with no known cure. The molecular and genetic mechanisms involved in HD are well understood, but available therapies mostly treat symptoms rather than addressing the underlying cause of this condition (56). In addition to chorea, patients with HD often experience other neurological issues, such as depression and psychosis. Various neuroleptic drugs (e.g., Acepromazine, Bromperidol, Benperidol) are used to manage chorea in HD patients (57). For those with seizures, anticonvulsants like valproic acid may be prescribed when, HD is associated with Parkinsonism, and then levodopa is often given. While no drugs yet exist to directly target the mutant Huntington protein, new disease-modifying therapies are under investigation (58). Recombinant adeno-associated viruses have been harnessed as vehicles to deliver RNA interference (RNAi) therapies in preclinical HD models, showing some promise in slowing disease progression. Another strategy is to implant cells secreting ciliary neurotrophic factors into the brain, and some benefits have also been reported in HD models (59). Future therapies that are neuroprotective and disease-modifying, including novel delivery paradigms by which drugs could be targeted to the CNS to destroy the mutant Huntingtin protein and to arrest disease progression are urgently needed.

## 4 Targeting blood-brain barrier by nanoparticles

**Figure 2:**
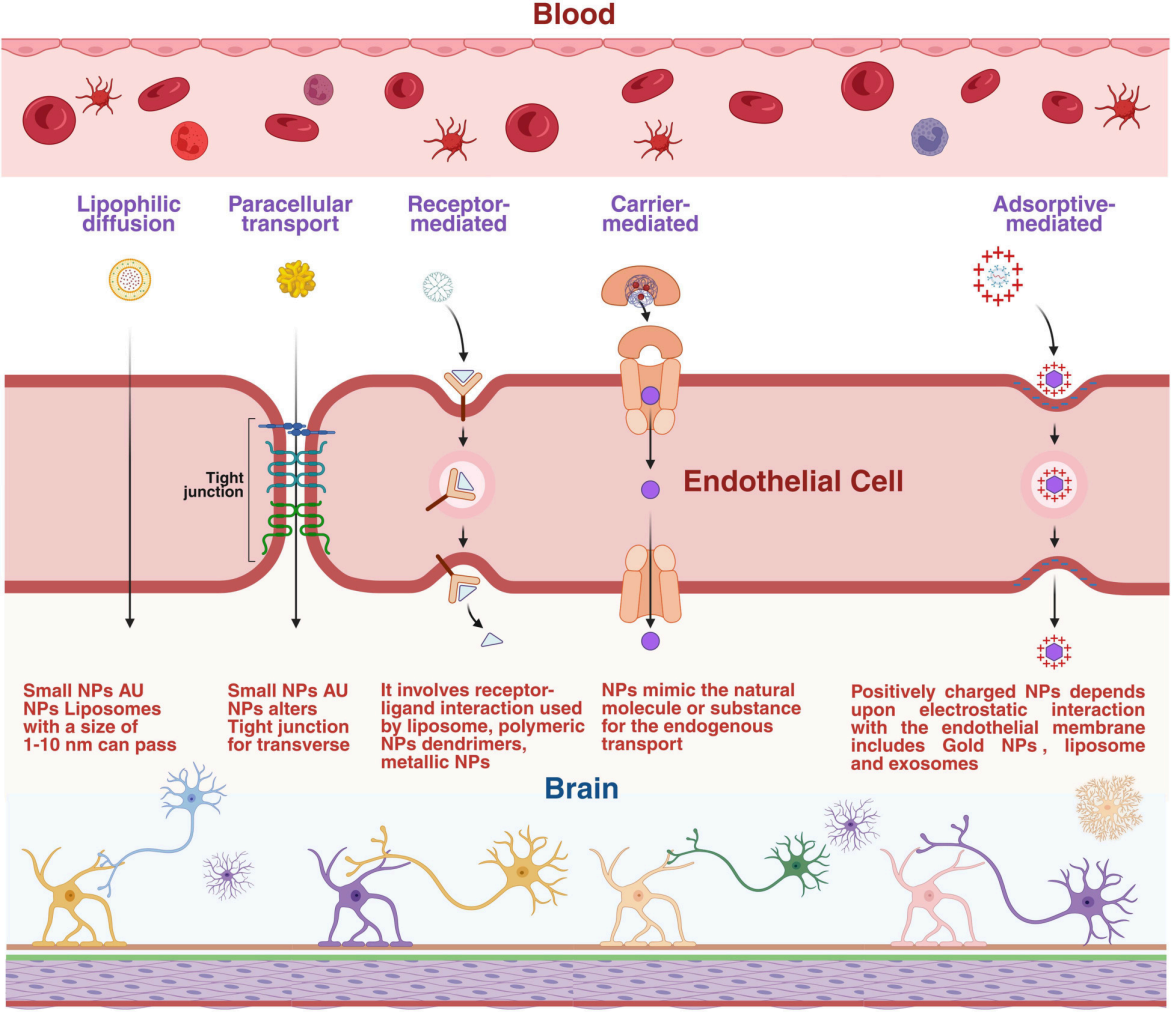
Transport of nanoparticles across the blood brain barrier. The diagram illustrates several pathways by which nanoparticles traverse the blood brain barrier (BBB). The BBB is formed by tightly linked endothelial cells which limits the entry of most molecule into the brain, NPs employ many strategies to cross the BBB, such as lipophilic diffusion, paracellular transport, receptor-mediated transcytosis, carrier mediated transport, and adsorptive mediated transcytosis. Once across the BBB these nanoparticles release their payloads into the neural tissue. Created in BioRender. Sharma, S. (2025) https://BioRender.com/esuy1ev

The blood-brain barrier (BBB) functions include supplying the brain with nutrients, protecting it, and preserving ionic equilibrium to maintain brain architecture and integrity. It protects the brain from neurotoxins coming from outside, and controls the levels of neurotransmitters (60). BBB is also responsible for preventing the macromolecules and poisonous chemicals from entering the brain (61). It controls the entrance of medications and governs the therapeutic efficacy of CNS treatments by providing or restricting entry to the CNS. The BBB is responsible for regulating the transfer of water and salts from the blood into the extracellular fluid, which maintains the consistent volume of the brain (62). Water and salts can easily penetrate brain tissue if in case the BBB is damaged due to mechanical injury or illness (13) leading to a rise in intracranial pressure and edema (63). The brain has high demands of nutrients and energy, despite the fact that the BBB places a significant amount of restrictions on the movement of substances across it, because of this, the brain is able to absorb endogenous chemicals through a variety of both passive and active transport pathways (13). Passive diffusion, carrier-mediated transport, and adsorptive-mediated transcytosis are the major mechanisms that allow substances to move through the blood-brain barrier (Figure 2).

### 4.1 Passive diffusion

Small nanoparticles move down their concentration gradients by a mechanism of transport known as passive diffusion, which is a nonspecific and energy-free mode of transport (64). Small molecules and nanoparticles that are water-soluble are able to permeate the blood-brain barrier (BBB) by the process of paracellular passive diffusion, which involves sliding down an inverse concentration gradient across tight junctions. The lipid soluble nanoparticles are able to pass through the membrane of endothelial cells and reach the blood-brain barrier (BBB) using a cross-cellular pathway (65).

### 4.2 Efflux pumps

ATP hydrolysing proteins embedded in the endothelial membranes also facilitate the transfer of molecules across the BBB using the energy generated from ATP breakdown. Since they work using energy they can transport substances against the concentration gradients, some efflux pumps present on the BBB are P-glycoprotein (P-gp), the breast cancer resistance protein and various drug resistance—associated protein, organic anion transporters, organic anion transporting polypeptides, etc. (66).

### 4.3 Transcytosis mediated by receptors

The Receptor-Mediated Transcytosis (RMT) acts upon the binding of nanoparticles to specific receptors on the luminal side of vascular endothelial cells (67). The binding causes membrane invagination, which is then followed by endocytosis. As the receptors for elements like iron, insulin, and leptin are extensively expressed in the lumen, these elements can get transported via RMT (68).

### 4.4 Transcytosis mediated via adsorption

The glycocalyx, which is made of heparan sulfate proteoglycans, imparts negative charges to the endothelial cell membrane of the BBB (69). In addition, the presence of sialo glycoproteins and sialo glycolipids contributes to the negative charge that is present on the surface of the BBB. Therefore, the attraction between positively charged cationic nano-molecules and the negatively charged surface of the membrane, can work to facilitate the transport of these nano-molecules into the brain (70).

### 4.5 Transcytosis by carrier's proteins

Transportation is facilitated by carriers and can be separated into two categories: secondary active transport and facilitated transportation. This particular mechanism is capable of transporting a variety of nanoparticles. In this mode, the nanoparticle initially affix itself to a specific transporter that is situated on the lateral aspect of the canal lumen (71) the transporter then undergoes a conformational change eventually transporting the NP into the brain.

## 5 Targeting specific regions in the brain by nanoparticles

Targeting specific regions of the brain for drug delivery has become increasingly important due to varying susceptibility of different brain areas in each neurological disease. One key strategy for targeting nanoparticles (NPs) to specific brain regions is by exploiting the alterations in the neurovascular units and the resulting changes in the local microenvironment associated with specific CNS disorders (72). For example, after a traumatic brain injury (TBI), thrombin influx into the injury site creates a localized hotspot that can be used to attract NPs that are modified with a peptide (CAQK) capable of binding specifically to thrombin in the affected area. Likewise, stroke-targeting peptides (e.g., CLEVSRKNC) bonded to NPs have also been effectively utilized to deliver neuroprotective drugs to stroke-injured sites in preclinical studies, resulting in decreased infarct size and improved recovery (72). Additionally, changes in the microenvironment, such as redox state, altered pH, and the presence of reactive oxygen species (ROS) at the pathophysiological sites, can act as sensitive stimuli to trigger drug release in those areas. For example, NPs sensitive to oxidative stress have been shown to accumulate in the wound area after TBI and promote functional recovery (73). Furthermore, certain chemical coatings on NPs have demonstrated a preference for accumulating in specific brain regions following systemic administration. For example, while PS80-coated Poly –lactic-co-glycolic acid (PLGA) NPs favoured cortical accumulation, the mechanisms involved in this targeting have yet to be elucidated. More research is required to unveil existing materials that can display such region-specific accumulation. Another important factor can be external stimuli like light, magnetic fields, and ultrasound, which have been shown to be effective in directing NPs to desired locations inside the brain (74). The shape, coating density, and light responsiveness of the NPs are all parameters that can be controlled for targeted accumulation. For example, upon transdermal delivery, transferrin coated gold nanorods preferentially localise to the hippocampus and subventricular zones in healthy mice upon near infrared light (NIR) activation. Similarly, super paramagnetic iron oxide NP-labeled mesenchymal stem cells have been guided to the hippocampus by magnetic fields in AD models (75). Nonetheless, guiding NPs to reach the entire area affected by the pathology without spreading into adjacent healthy tissues is still problematic, often requiring extensive optimization of NPs physiochemical properties (size, charge, stiffness, etc.) (76). Advances in single-cell omics and sequencing technologies have made it possible to uncover cellular and molecular signatures in health and disease in the brain that are distinct at the single cell level. Moreover, the selective vulnerability of specific cell types and the spatiotemporal patterns of degeneration both highlight that targeted delivery approaches will be a necessity to maximize treatment efficacy and minimize unwanted effects and toxicity.

## 6 Targeting specific cell types in the brain by nanoparticles

Astrocytes play a crucial role in maintaining cerebral homeostasis by regulating the BBB, recycling neurotransmitters, remodelling synapses, and balancing ion concentrations (77). Several studies have exploited ligand-conjugated NPs (ligand-NPs) for the specific targeting of astrocytes. Targeting astrocytes is critical for addressing neurological disorders in the CNS, because of their structural proximity to the brain endothelium and the BBB's disruption in various NDs diseases. For instance, an arginine-rich gene delivery system selectively transfected primary human astrocytes, with limited localization in neurons (78). Likewise, LPS-functionalized NPs were used to specifically target reactive astrocytes for spinal cord injury model systems, although some neuronal uptake was observed as well. Similarly, other ligands (triphenylphosphonium, AS1) and gH625 (viral derived peptide for effective delivery) have already been shown to preferentially target astrocytes (79). Additional investigation on the moderated expression of receptors and transporters during disease states could aid in better optimization of such approaches.

Microglia, the brain's resident immune cells, eliminates dead cells, and debris, secretes neurotrophic factors, and contributes to the overall homeostasis of the CNS (80). Microglial phenotypes and genotypes are highly heterogeneous based on the disease state itself, and this poses considerable challenges and opportunities for targeted delivery. However, microglia tend to interact more strongly with spiked nanostructures than with spherical or rod-shaped nanoparticles (81). Similarly, LPS-liposomes targeted to TLR4 receptors accumulated preferentially in microglia and delayed disease progression. In chronic neuroinflammation, microglia-targeting lipid nanoparticles (MG-LNPs) with siRNA payload have shown the therapeutic delivery of their payloads, as evidenced by improvements in reducing inflammation even when administered through non-optimal routes. NPs have also targeted other microglia-specific receptors such as scavenger receptors, receptors for advanced glycation end products (RAGE), and cluster of differentiation (CD) receptors (82), improving uptake *in vitro* and calling for more such investigations *in vivo*.

Both functionally and morphologically, neurons look entirely different in various regions of the brain and under different disease states. This diversity makes neurons selectively vulnerable in various NDs making it crucial to target neurons using more specific and precise methods. Both the shapes and sizes of nanoparticles can influence how neuronal cells regenerate themselves, becoming either differentiated or undifferentiated culminating in life-or-death issues bringing the focus to nanoscaffolds, nanotubes, and nanowires (83). Nano-scaffolds facilitate the growth of cells and tissues. Nanotubes are nanometer-sized cylinders, while nanowires are ultrathin, wire-like structures with diameters of 1–100 nm. Smaller nanoparticles, such as 15-nm gold NPs, have been shown to be taken up preferentially by neurons-rather than larger ones (84). In addition, the surface charge of nanoparticles is crucial for neuronal targeting. For example, negatively charged nanoparticles interact selectively with neuronal membranes in comparison with positively charged or uncharged ones (85).

Some nanoparticles have been designed to target neurons more specifically, such as gold NPs coated with exosomes that carry Rabies Virus Glycoprotein (RVG) peptide and lysosome-associated membrane protein 2b (RVG-Lamp2b) fusion peptides (86), which targets nicotinic acetylcholine receptors on neurons. While RVG facilitates neuronal specificity, astrocytes and microglia can also express these receptors under pathological conditions, raising the need for caution in targeting. Another neuronal-targeting peptide, Ten Eleven Translocation one (Tet-1), derived from tetanus toxin, binds to tri-sialo-ganglioside receptors located on the surface of motor neurons and has been used successfully to deliver therapeutic agents to neurons in several models (87). Ligands such as nerve growth factors (NGFs), dopamine, transferrin (TF), and lactoferrin have additionally been investigated to target certain neuronal populations that partake in diseases like Alzheimer's and Parkinson's (88). As an example, silica nanoparticles conjugated with dopamine selectively deliver antioxidants to dopaminergic neurons *in vitro* (89). However, more studies are needed to assess the relevance of *in vivo* targeting of specific neuronal subtypes, including dopaminergic, glutamatergic, and GABAergic neurons, highlighting the need to develop this approach for receptors that exhibit dynamic expression in NDs. To enable selective and efficient cell type-specific drug delivery in the brain, we need to understand its cellular and molecular landscape in health and disease. Consequently, optimization of the strategies for more precise targeting of astrocytes, microglia, and neurons is much needed to develop the best possible therapies with the least neurotoxicity (90).

## 7 Recent advances and clinical trials for neurodegenerative diseases

### 7.1 NP-based siRNA delivery for ADs

The instability of siRNA therapy in the circulation and the fact that it is unable to reach target cells in the central nervous system (91, 92) because of the BBB poses the biggest hurdle in siRNA therapy. With technological advancements, it is possible to design nanoparticles to be able to traverse the blood-brain barrier, increase cellular absorption, and give protection against enzymatic degradation, all of which make them an attractive candidate for delivering siRNA to the brain. Nanoparticles utilized for this purpose include Lipoprotein-based nanoparticles, Polymeric nanoparticles like polylactic-co-glycolic acid (PLGA) or polyethylenimine (PEI) etc. LNPs, which encapsulate siRNA within lipid bilayers, have received the most attention from researchers (93) owing to the fact that they have the ability to protect siRNA and facilitate efficient endosomal escape (94). To facilitate targeted transport to neuronal cells, LNPs can be functionalized with ligands such as transferrin or rabies virus glycoprotein (RVG) peptides (95). Furthermore, they can be further modified using polyethylene glycol (PEG) in order to boost their stability. Polymeric nanoparticles, such as those generated from polylactic-co-glycolic acid (PLGA) or polyethylenimine (PEI), also appear promising, as these materials can be tailored to deliver siRNA precisely to microglia or neurons while allowing for prolonged release (96).

Nanoparticles delivery systems have now emerged as a powerful strategy for facilitating the passage of siRNA over the blood–brain barrier for AD therapy (97, 98). For instance, in transgenic AD animal models, Lipid Nanoparticles have been employed to deliver siRNA that targets the amyloid precursor protein (APP) or presenilin 1 (PSEN1). Such treatment has resulted in a substantial reduction in the accumulation of amyloid-β (Aβ) plaque and an improvement in cognitive performances (99, 100). In order to decrease the amount of tau phosphorylation and the formation of neurofibrillary tangles, siRNA against microtubule-associated protein tau (MAPT) has been shown to have a protective effect on synapse function and neuronal survival (101).

Targeting the APOE ε4 allele or inhibiting TREM2 in microglia, leads to clearance of Aβ, decreasing the inflammation of the nervous system, and slowing AD progression (102).

The delivery of siRNA by nanoparticles has been examined on genes associated with immune response, lipid metabolism and genes coding receptors expressed on myeloid cells 2 (TREM2) and apolipoprotein E (APOE) (103, 104). The siRNA-NP targeting diverse molecular mechanisms implicated in Alzheimer's disease, to downregulate genes such as bridging integrator 1 (BIN1), sortilin-related receptor 1 (SORL1), and clusterin (CLU) has proven useful in various animal models (105).

Molecular structures of siRNA are subjected to chemical modifications, such as the addition of 2′-O-methyl or 2′-fluoro replacements (106), in order to enhance their stability and reduce the occurrence of off-target effects, while simultaneously enhancing transport efficiency and limiting adverse effects. The Nanoparticle platform's unique siRNA sequence delivery also allows it to target numerous AD pathogenic pathways simultaneously (107, 108).

Min et al. employed the GLUT1 transporter to facilitate the delivery of antisense oligonucleotides (ASOs) by designing GLUT1-targeted ASO-loaded glucosylated-polyion complex micelles (Glu-PIC/Ms) in mice models. The various glucose-modified PIC/Ms improved brain by-products owing to their multivalent interaction with GLUT1 located on the brain capillary endothelial cells (109) siRNA loaded magnetic nanoparticles have been effectively used as delivery mechanisms for targeting microglia in AD to reduce neurodegeneration (110). Barbosa et al. devised a delivery mechanism for siRNA to address AD by developing PEGylated magnetite nanoparticles encapsulating siRNA. In this study the BACE1 gene was targeted for *in vitro* RNA interference using HF-1 cells (111). The results demonstrated a substantial decrease in BACE1 levels without any kind of neurotoxicity, thus presenting an alternative for optimal siRNA delivery in gene therapy for AD. A Trojan horse technique to facilitate BBB penetration of stabilized glycosylated polymeric siRNA nanomedicine (Gal-NP@siRNA) in transgenic mice model was explored (97). The siRNA successfully targeted BACE1 and decreased the expression significantly (112). The growing body of evidence from *in vivo* research indicates that nanoparticles based siRNA therapies have proven effective as targeted AD therapeutics (99). Nevertheless, before these technologies may be clinically translated, there are a number of challenges that need to be conquered. These challenges include ensuring consistent BBB penetration, preventing immunological activation, achieving precise gene silencing, and demonstrating long-term safety. Nanotechnology and RNA interference offer promising ND treatments, as the combination can provide precision medicine based on each patient's molecular profile (113). Moreover, ongoing advancements in gene targeting, nanoparticle engineering, and biomaterial safety will be essential for advancing this therapeutic strategy from preclinical models to clinical trials.

### 7.2 NP-strategy for ALS therapeutics

**Figure 3:**
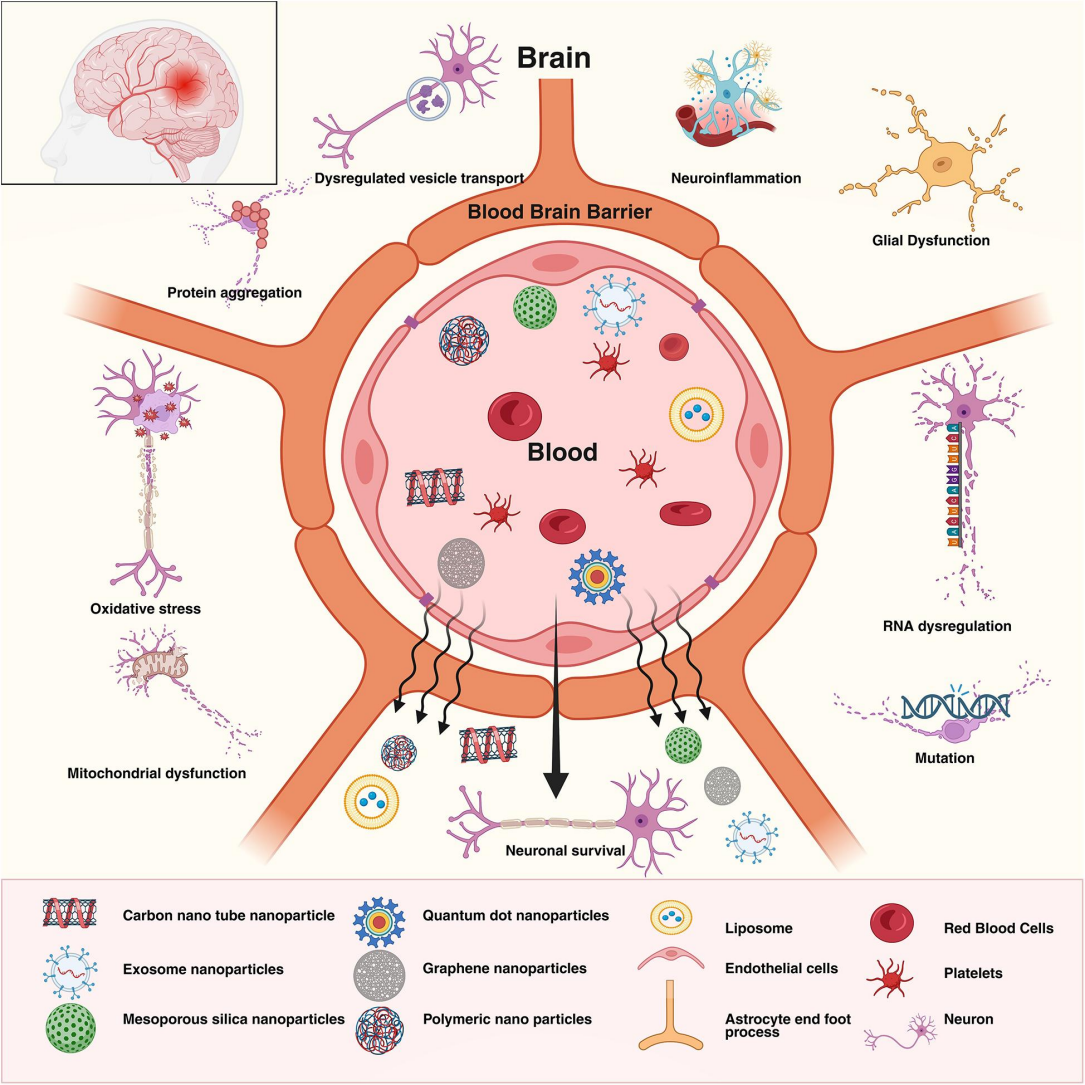
ALS pathophysiology and therapeutic potential of nanoparticles. The diagram illustrates the different pathophysiology of ALS highlighting the critical pathogenic features in the motor neurons and adjacent brain tissues. Various nanoparticles and blood cells have been shown in the blood vessel lumen, and the adjoining astrocyte end foot processes, which eventually traverse the BBB. The targeted delivery of nanoparticles carrying drugs or active pharmaceutical molecules promotes neuroprotection by improving neuronal survival. Created in BioRender. Sharma, S. (2025) https://BioRender.com/rhrmr16

A promising approach is to improve drug delivery for ALS through targeted, nano-enabled drug delivery systems, such as polymeric nanoparticles (114). So far, only a handful of studies have investigated the development of targeted nanotechnology for ALS in animal models. As an anti-glutaminergic agent, riluzole is used to attack glutamate receptors (GluRs) to prevent any abnormal stimulations (115). In one study, riluzole-loaded solid lipid nanoparticles were developed, demonstrating improved BBB penetration and higher drug delivery to the brain compared to conventional riluzole. These nanoparticles also decreased drug deposition in peripheral organs, thus substantially lowering the side effects (116) (Figure 3).

A different study made use of carbon nanotubes (CNTs) to target drug delivery of riluzole, where CNT characteristics for ideal drug delivery, were optimized and it was observed that the CNTs were non cytotoxic *in vitro* (116, 117). Mesoporous silica nanoparticles (MSNs) were used for treating an ALS in an animal model with a mix of drugs, including leptin (which protects nerves) and pioglitazone (which reduces inflammation) (118, 119). Graphene quantum dots (GQDs) were helpful for treating the ALS, that is related to proteinopathy, by changing how TDP-43 aggregates (120). The C-terminus of TDP-43 has an intrinsically disordered area that can produce amyloid fibrils. However, GQDs stopped the development of amyloid fibrils by directly interacting with TDP-43 (121). In another research, the chitosan nanoparticles were exploited, because of their hydrophilic nature and cationic properties, which allowed them to cross the BBB and have a high drug entrapment and release efficiency (122). Targeted delivery systems were also explored for other agents, such as minocycline (123). Targetted formulation of lipid-coated minocycline, which preferentially accumulates in microglia of (SOD1) ALS mice; demonstrated a 29% higher drug uptake than non-targeted liposomes and a superior impact on disease delay when delivered by the intracerebroventricular route in ALS mice (124, 125). Polystyrene nanoparticles (PS) cause aberrant condensation of TDP-43 and have been used as therapeutics for ALS (126). When cells are exposed to PS, they go through oxidative stress, which causes TAR DNA-binding protein 43kDa (TDP-43) to clump leading to ALS-like symptoms. Also, the oxidized form of heat shock protein 70 is incapable of transporting TDP-43 back to the nucleus. The buildup of TDP-43 in the cytoplasm makes it easier for PS and TDP-43 to form a complex that clumps and hardens with time (127). Their trials though appeared promising but could not be extrapolated for humans, showing the urgency for novel models to test nanobased ALS therapies (Figure 3). Still, the exosome delivery methods should be able to move quickly to clinical trials because they are well-tolerated by living things (128), so, drug conjugated exosomes for deliveries over the BBB are increasingly explored for ALS and other neurological illnesses (129).

### 7.3 Extracellular vesicles (EVs) as innovative drug delivery systems

Exosomes are small, ranging in size from 30 to 150 nm, and originate from biological sources. Exosomes could be useful in treating a wide range of medical conditions, such as infections, cancer, neurodegenerative disorders, and tissue repair (130–132). Researchers are using methods like sonication, electroporation, and surface chemical modification to systematically design EVs that can carry proteins, nucleic acids, and small pharmaceutical compounds for a variety of medical uses (133). The brain cells, including neurons, oligodendrocytes, astrocytes, and microglia, use the EVs for vital communication that affects synaptic connections, neural growth, and cell differentiation (134). Extracellular vesicles can traverse the blood-brain barrier and facilitate communication among various cell types, undoubtedly, this property is a distinct EV characteristic that is unparalleled to conventional drug carriers (135, 136). EVs are exceptional drug delivery vehicles and have emerged as powerful alternatives for tailored distribution of drugs to treat neurological diseases, as they are biocompatible and stable in circulation for several days (137, 138). In, CNS affected patient, EVs are used for administering drugs directly to the brain (139) and for deliveries across the blood-brain barriers (140). Also, the immune system is less likely to reject exosomes since they are swiftly absorbed by cells and can easily circulate throughout the body (141). They can be targeted with surface molecules like integrins and tetraspanins (142). For instance, EVs generated from breast cancer cells can transcytose into the intact blood-brain barrier (143), and were used therapeutically to target the brain (139). EVs produced by mesenchymal stem cells can promote the creation of new blood vessels and neurons, help repair neural tissue, modify immunological responses, and aid brain ischemia recovery (134, 144).

EVs are used to address multiple aspects of AD etiology, as studies confirm that EVs significantly decrease Aβ levels in murine models by binding Aβ to glycosphingolipids that are internalized by neurons. Conversely, other research confirms that mesenchymal stem cells (MSCs) EVs are internalized by microglia and astrocytes. EVs for AD therapy are most often derived from Mesenchymal Stem cells (MSC). MSC-EVs function predominantly by the activation of autophagy via the catalase enzyme, the Nrf2 signalling pathway, the miR 146a-inhibited NF-κB pathway, and the targeting of the PTEN-PI3 K/Akt pathway by miR-223 (145). EVs have also successfully shown remarkable ability as a disease-modifying therapy for PD. MSCs-EVs penetrate the blood-brain barrier and provide neuroprotection by enhancing cell proliferation and suppressing apoptosis. Furthermore, miR-200a-3p enriched EVs derived from healthy astrocytes decline the expression of mitogen-activated protein kinase 4 (MKK4), a crucial kinase in the c-Jun N-terminal kinase apoptotic pathway (146).

Inferrings from the few studies of ALS therapy, it was seen that EVs from adipose stem cells, can safeguard SOD1 mutant neurons from oxidative stress and restored mitochondrial function. MSC-EVs were efficiently internalized by mouse BECs and restore BBB strength. MSC-EVs facilitated neurite growth and accelerated the level of antioxidant and anti-inflammatory genes (147). EVs can diminish mutant huntingtin (mHTT), a protein that is responsible for neuroinflammation in HD (148). EVs of adult stem cells and neural progenitor stem cells effectively restore mitochondrial activity reduce N-terminal cleaved mHTT and inhibit apoptosis. Furthermore, studies in a murine model demonstrated that EVs transported by blood can reduce mHTT levels and neuronal degeneration. Now, the exosomes are becoming significantly instrumental in the field of nanomedicine because they are efficient, safe, and posses the ability to pass through biological barriers with minimal harm (149, 150).

### 7.4 Lipid nanoparticles (LNPs) based ND therapeutics

Lipid-based nanocarriers are garnering significant attention due to their successful and highly effective application in the production of anti-COVID-19 vaccines (151–153). The potential of these nanoparticles lies in their ability to facilitate the passage of medications through the blood-brain barrier, thereby enhancing the effective delivery of treatments for neurological conditions (154, 155). The advantages of functionalized lipid nanoparticles encompass enhanced penetration and an extended residence time of therapeutic molecules at the blood-brain barrier (156–158). Moreover, lipid nanocarriers designed for active targeting have the potential to facilitate drug delivery to the brain (159–163). Lipid nanoparticles (LNPs) have the ability to encompass both hydrophilic and lipophilic compounds, emerging as encouraging drug delivery systems for improved active targeting and penetration into the BBB (164). Active targeting involves the attachment of ligands to the surface of nano systems (165, 166) to enhance their interaction with proteins that are consistently expressed at the blood-brain barrier, including the insulin receptor, transferrin (Tfr) (167), and low-density lipoprotein receptor (168, 169). Lipid nanoparticles can be characterized by their physico-chemical properties as niosomes, liposomes, solid lipid nanoparticles, nanostructured lipid carriers, nanoemulsions, and cubosome-type liquid crystalline nanoparticles. Different lipid nanoparticle types may alter brain medication delivery, including stability and release and selecting the right lipid nanoparticles for brain medication delivery is crucial (65). LNPs can be functionalized with transferrin or Angiopep-2, and bind to endothelial cell receptors and assist in crossing the BBB (170).

LNPs have been extensively studied for AD treatment and a brief account of the different studies conducted so far in AD mice models is presented here. Curcumin and donepezil were co-encapsulated in nanostructured lipid carriers using a micro-emulsion process and administered intranasally in mice. The *in vivo* pharmacokinetic investigations showed that the drug concentration in the brain was higher with Nano structured lipid carriers (NLC) as compared to the free medication given intravenously. NLC also resulted in a significant rise in acetylcholine (AChE) with a concomitant decrease in oxidative stress levels. Additionally, compared to the Wistar rats who were given the free medication, the experimental group showed improved memory and learning ability (171). A hydrophilic molecule Donepezil(DPL), has been endorsed by the US-FDA for AD therapeutics (172). The problem with DPL is its minimal BBB permeability and increase in the dose frequency leads to significant cholinergic adverse effects. DPL-loaded LNPs were produced by solvent emulsification diffusion method utilizing glycerylmonostearateas and surfactants like Tween® 80 and poloxamer 188 (1:1). *In vivo* tests were done by delivering DPL-LNPs via the intranasal route and DPL solution intravenously in male albino Wistar rats. The intranasal treatment with DPL-loaded LNPs resulted in a considerably high concentration of medication in the brain, showing better brain target delivery (173). An inhibitor of the enzyme Acetyl choline esterase, Galantamine (GH), too suffers from similar limitations like DPL (174). Glycerylbehenate (Compritol®) as the lipid, Pluronic® F-127 as surfactant, and Tween® 80 as co-surfactants were used for synthesizing GH-loaded LNPs by microemulsification (175). GH-LNPs proved better for *in vitro* drug release and *in vivo* memory repair potential in isoproterenol-induced cognitive challenged adult wistar rats (176).

To mitigate the limitations of existing Parkinson's disease medications, such as levodopa-induced dyskinesias, new compounds are being developed or are in the early stages of clinical trials to influence disease progression. Novel compounds and therapeutic approaches exhibit restricted clinical efficacy attributable to factors such as short half-life, *in vivo* stability, and challenges associated with blood-brain barrier penetration, among others (177). Researchers have conducted extensive studies on lipid-based nanocarriers, including liposomes, exosomes, nanoemulsions (NE), and solid lipid nanoparticles (SLN), to tackle the aforementioned issues. Recently, phospholipid-based gelatin nanoparticles containing basic fibroblast growth factor (bFGF) made with the non-ionic copolymer poloxamer 188 have been tried in hemiparkinsonian rats. The poloxamer 188-induced transcellular transport increased bFGF levels in the striatum and olfactory bulb and enhanced nasal mucosa lipid bilayer penetration. The carriers’ strong surface negative charge also prevented mucociliary clearance and extended residence time, which enhanced drug absorption. The study indicates that LNPs could be employed as drug delivery vehicles to transfer genes across the blood-brain barrier (178).

Another work used melt emulsification to encapsulate GDNF and make chitosan (CS)-coated NLPs (179) using TAT, transactivator of transcription (TAT), a cell penetrating peptide (CPP). Intranasal injection in a 1-methyl-4-phenyl-1,2,3,6-tetrahydropyridine PD mouse models was done to assess the *in vivo* performance of these carriers. The CS-NLPs-TAT-GDNF group recovered motor function at two weeks which was much earlier than the control group. The work demonstrated promising results which could be extrapolated for future clinical trials (180). Studies have suggested that lipid-based antioxidant nanocarriers can reduce oxidative stress-induced neuroinflammation. Research has examined the LNPs of idebenone (IDE), gold NP of lipoic acid, liposomes and NE of resveratrol, NE of vitamin E and coenzyme Q10, and NE selegiline (181). These nanocarriers were useful in addressing bioavailability difficulties caused by short half-life, substantial first-pass hepatic metabolism, and poor BBB penetration (182). The flavonoid Vitexin from *Crataeguspinnatifida* Bunge, was conjugated to LNP for testing in a 6- hydroxydopamine (6-OHDA) induced PD animal model (183). The vitexin-loaded LNPs enhanced total reactive antioxidant activity (184) and was potent in preventing memory loss in Morris water maze and depressive-like behaviour in tail suspension tests (185). Even though LNPs have enhanced CNS targeted delivery research, none have been approved for commercialization after completing all clinical trials. Although lipid-based carriers have many benefits, their translational development into clinical practice must be accelerated (186). With the advancing proteomic, lipidomic, and genomic technologies, effectiveness of hybrid LNCs could be increased to achieve scalability and create affordable, effective dosage forms (98).

### 7.5 Clinical trials in FDA pipeline

Although several unique properties of nanoparticles make them highly lucrative for focused medical applications (187) yet clinical trials are necessary to ensure safe, effective therapies and for medical improvement (188). Clinical trials involve rigorous testing on human subjects to determine the drug's effectiveness and potential side effects, providing accurate data for clinical decision-making, regulatory approval, and scientific research. A recent update of ongoing and completed nano-carriers in the FDA pipeline for the treatment of various NDs is detailed in Table 3. ClinicalTrials.gov has assigned the study number NCT03806478 to the APH-1105 study, which is a Phase 2 clinical investigation that was launched in 2019. Patients aged 50 and older suffering from mild to severe AD were enrolled. The study was sponsored by Aphios Corporation and to investigate the effects of intranasal APH-1105, an alpha-secretase modulator. Approximately 60 individuals were included in this randomized, triple-blind, placebo-controlled experiment and were given doses ranging from 0.5 µg, 1.0 µg, and 2.0 µg, as well as a placebo group. Utilizing instruments such as the ADAS-Cog and the Hopkins Verbal Learning Test, the primary objective of the study was to evaluate the cognitive improvements, as well as the safety and tolerability of the treatment (https://clinicaltrials.gov/study/NCT03806478?term=NCT03806478&rank=1).

**Table 3:** Clinical trials of nanoparticles for treatment of neurodegenerative diseases.

Neurodegenerative disease	Therapy	Nanocarrier type	Clinical stage	Trial ID
Alzheimer's (mild to moderate)	APH 1105	Liposomal formulation	Phase II (planned)	NCT03806478 (Not yet recruiting)
Alzheimer's disease	ACI 35.030 & JACI 35.054	Liposomal nanoparticles	Phase II (ongoing/planned)	NCT04445831; (tau-targeting liposomal vaccines by AC Immune SA)
Parkinson's Disease	CNM Au8	Gold nanocrystals (catalytic)	Phase II (completed)	NCT03815916 (REPAIR PD)
Parkinson's disease	2B3 101 (Talineuren)	PEGylated Glutathione liposome (encapsulating doxorubicin)	Phase I (ongoing)	NCT04976127 (enhanced BBB penetration)
ALS	CNM Au8	Gold nanocrystals (catalytic)	Phase II (completed)	NCT04098406 (ALS)
ALS and MS	CNM Au8 (Expanded Access)	Gold nanocrystals (catalytic)	Expanded-access/open-label safety study	NCT04081714 – MGH-led safety, PK/PD

The Phase 1b/2a clinical trial (NCT04445831), which was sponsored by AC Immune SA (clinical-stage biopharmaceutical company), examined the safety, tolerability, and immunogenicity of two tau-targeted liposomal vaccines, namely ACI-35.030 and JACI-35.054, in the early stages of AD. The randomized, double-blind, placebo-controlled trials were conducted over a period of 48 weeks and involved numerous intramuscular doses provided over several weeks and long-term safety monitoring was performed until week 74. Interim findings showed that anti-phosphorylated tau (pTau) IgG responses were robust across all dosage levels, with titres rapidly increasing and remaining stable after the boost. It was demonstrated that both vaccines are safe and well tolerated, and there have been no dose-limiting toxicities or significant adverse events that have been ascribed to treatment (https://clinicaltrials.gov/study/NCT04445831?term=NCT04445831&rank=1).

The effect of CNM-Au8, a colloidal suspension of catalytically active clean-surface gold in early PD, was evaluated under the Phase 2 design for trial number NCT03815916. The research was carried out by Clene Nanomedicine, and involved the participation of 13 individuals who had been diagnosed with PD. The primary purpose of this study was to evaluate the modification of neuronal redox state through the utilization of ^31^Phosphorus Magnetic Resonance Spectroscopy (^31^P-MRS), with a particular emphasis on the ratio of NAD^+^ to NADH in the brain. Safety, tolerability, and exploratory indicators of mitochondrial functioning were included among the secondary objectives (https://clinicaltrials.gov/study/NCT03815916?term=NCT03815916&rank=1).

Another 2021 open-label, single-center Phase I trial NCT04976127 looked at the safety, tolerability, and pharmacokinetics of intravenous TalineurenTM (TLN), a liposomal GM1 ganglioside for PD. Twelve people were enrolled for eight weeks, and dose response established the highest dose at 720 mg. Some not so serious side effects were reported, and seven patients had brief infusion responses after getting TLN. The pharmacokinetic study found a C_max of 4 h and a terminal half-life of 12.6 h, and statistically significant improvements in motor (MDS-UPDRS), non-motor (NMSQuest) functions, as well as in the quality of life were observed. (https://clinicaltrials.gov/study/NCT04976127?term=NCT04976127&rank=1).

Another, Phase 2 randomized, double-blind, clinical trial that examined the efficacy and safety of CNM-Au8 in subjects having early symptoms of ALS (NCT04098406) and already receiving steady background therapy, such as riluzole was conducted. The patients were given either CNM-Au8 or a placebo and alterations in motor neuron function and respiratory metrics were included as primary objectives, whilst biomarkers of neurodegeneration and quality of life evaluations were included as secondary outcomes. Although the trial did not reach its primary effectiveness goals, exploratory analysis suggested that CNM-Au8 might play a role in delaying clinical worsening. This was demonstrated by a sixty percent reduction in mortality risk when compared to the placebo therefore subsequent trials to determine whether or not CNM-Au8 could provide neuroprotective advantages in ALS and other neuro-degenerative disorders are urgently required (https://clinicaltrials.gov/study/NCT04098406?term=NCT04098406&rank=1).

CNM-Au8, nanocrystals were also tested on patients of relapsing-remitting multiple sclerosis (RRMS), non-active secondary progressive multiple sclerosis (SPMS), or primary progressive multiple sclerosis (PPMS) in an investigator-initiated, open-label, expanded-access study (NCT04081714) for evaluating the safety, pharmacokinetics (PK), and pharmacodynamics. Serial assessments included clinical laboratory measures, efficacy measures, and biomarkers associated with neuroprotection and remyelination to assess the therapeutic potential of CNM-Au8 were accomplished (https://clinicaltrials.gov/study/NCT04081714?term=NCT04081714&rank=1).

## 8 Potential toxicological risks and immunogenicity associated with nanoparticles

**Figure 4:**
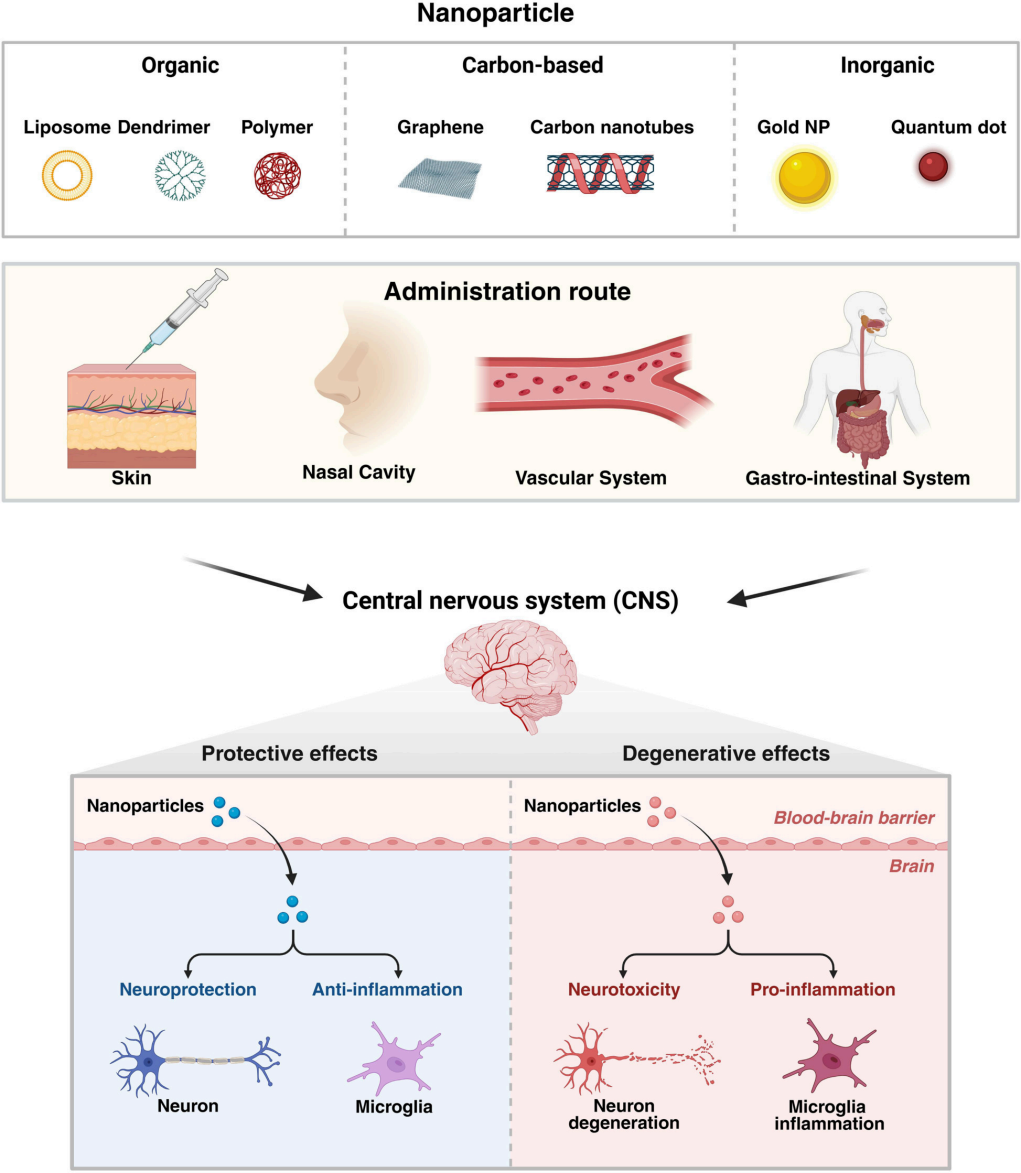
Nanoparticles and their administration routes for ND therapeutics. The diagram illustrates the type of nanoparticles and their possible administration pathway, which includes skin, nasal cavity, and the vascular and gastrointestinal systems, for delivery into the central nervous system. Upon crossing the blood-brain barrier, nanoparticles can cause either protective effect, including neuroprotection and anti-inflammatory responses benefiting the neurons and microglia, or degenerative effects, including neurotoxicity and pro-inflammatory reactions, which result in neuronal degeneration and microglial inflammation. Created in BioRender. Sharma, S. (2025) https://BioRender.com/9jitye1

Physicochemical properties of the NPs provide significant advantages in a wide range of fields, particularly in medicine and industry (189) owing to their compact size, huge surface area, and substantial surface reactivity (190). The engineered nanomaterials (ENMs) pose major risks to the environment as well as for humans and other animals (191). The toxicity of these substances can be influenced by numerous factors like the size, shape, composition, surface charge, coatings, and phase stability of the particles (192). Nanoparticles pass through biological barriers and interact with cell structures, which may cause either short-term or long-term damage (Figure 4) (193).

Studies have shown that nanoparticles have the ability to have a negative impact on a range of human systems, including the nervous system (194). This is because nanoparticles have the ability to reduce the viability of cells and interfere with the processes that are regularly carried out by cells. The bio- distribution and toxicity of nanoparticles include surface modifications and the formation of protein coronas in biological environments (195, 196). Toxicological mechanisms that are considered to be among the most significant include the generation of reactive oxygen species (ROS), oxidative stress, inflammation, genotoxicity, and cytotoxicity (197–199). After entering an organism, NPs may disperse throughout its tissues in ways that are not controlled by established physiological mechanisms. Because of the complex relationships between biological systems and nanomedicines, there is currently no clear advice for evaluating nano-toxicology using conventional toxicological methods. Experts and toxicologists have not agreed on a standardized protocol for toxicological testing and analysis, as it necessitates a multidisciplinary approach that incorporates environmental science, chemistry, physics, toxicology, materials science etc.,.

To evaluate the toxicity of nanoparticles, it is necessary to conduct a complete study that makes use of both *in vitro* and *in vivo* approaches (200). The widespread use of *in vitro* techniques is due to the fact that they are very cost-effective and ethical. These procedures include using tests like measuring gene expression, cell viability testing, hemolysis, and genetic damage. To characterize NP interaction with cells at the microscopic level, techniques like transmission electron microscopy (TEM), scanning electron microscopy (SEM-EDX), atomic force microscopy (AFM), and spectroscopic methods can be used. *In vitro* tests are good for initial screening, but *in vivo* tests are still more reliable because they provide insights at absorption, circulation and clearance of nanoparticles (201–204). With the ever-increasing use of NPs it is absolutely necessary to have a comprehensive understanding of the toxicological impact that these substances have in order to ensure the safety of both people and the environment. A more comprehensive regulatory framework grounded in advanced scientific principles and characterized by transparent communication across all sectors is of the utmost necessity, to ensure the safety, efficacy, and sustainability of nanotechnology in healthcare.

## 9 Expert commentary

NPs platforms have the ability to revolutionize the delivery of therapeutics to the CNS due to their tuneable size, shape, surface charge, and ability to conjugate any number of ligands (205). This versatility provides a potential means of targeting discrete structures within the brain with such specificity. The development of nanoparticle-based drug delivery systems, such as lipid-based nanoparticles, extracellular vesicles, and functionalized nanoparticles, has significantly improved active targeting in the treatment of neurodegenerative diseases. Solid lipid nanoparticles and liposomes are being extensively investigated as potential therapeutic agents for Alzheimer's and Parkinson's diseases. These nanoparticles exhibit high precision in targeting specific brain sites and can transport both lipophilic and hydrophilic molecules into the brain. With the development of extensive production capabilities, the mRNA vaccines, conjugated with LNPs, will soon emerge as highly promising clinical candidates for the treatment of Parkinson's and Alzheimer's diseases in the near future. Polymeric nanoparticles employing polylactic acid (PLGA) carriers have also shown notable stability and controlled release of contents, indicating substantial potential in preclinical models of Huntington's disease.

At this time point, although dendrimers and inorganic nanoparticles demonstrated potential for theranostics and drug administration; however, their toxicity and clearance challenges reduce their applications for NDs. Nanostructured lipid carriers and solid lipid nanoparticles can provide long-term release in the treatment of multiple sclerosis and PD, yet they aren't as flexible as polymer or exosome-based systems. Exosomes and biomimetic nanoparticles, natural or synthetic vesicles can be targeted to certain cells and are very efficient in transporting siRNAs, mRNAs, and small molecules across the blood-brain barrier.

If the pros and cons of NPs available for ND is weighed then the lipid nanoparticles are the safest and easiest for productions on a large scale, so they are best for short-term use while exosomes and biomimetic platforms, on the other hand, are the most dependable and efficient ways to deliver drugs to the CNS over time. Undeniably, the future of nanotherapeutics would be of hybrid nanocarrier systems that would combine the benefits of two or more NPs integrated into a single NP form for better targeting and treatment of NDs. However, significant progress is needed to translate the use of NPs for the treatment of CNS diseases.

The pace of this progress can be accelerated by integrating advanced tools like machine learning, high throughput screening, and single-cell RNA sequencing which can facilitate optimizing the design of these nanoparticles and their functionalization (206). These strategies can potentially aid experimental design, especially if *in vivo* phage display is used to discover homing peptides that can traffic to the brain (207). Moreover, integrating the recently developed 3D models of BBB into multiscale physiologically based cancer models offers better reflections of cellular dynamics than previous models, thus providing improved predictions of nanoparticle behaviour in the brain. In addition, technologies such as RNA sequencing, and spatial transcriptomics may improve our understanding of the molecular identities of different cell types in the brain (208) which can pave the way for designing more efficient drug delivery systems that are tailored to specific states of disease. Nonetheless, more research needs to be done to elucidate NP interactions with the diverse regions of the brain and the ways in which these cell-cell interactions change under disease conditions, especially with the increasingly detailed maps of brain cell functions and interactions that are now available. Elucidating the complexity of neural communication networks, including the interactions between neurons and glia, can yield far-reaching consequences, presenting opportunities and solving conundrums for precise targeting (209).

NPs can provide a means for delivering therapeutic entities that can modify mitochondrial metabolism and bioenergetics, thereby addressing one of the fundamental aspects of the pathology of the disease (210). NPs can be designed to engage certain brain cell receptors, thereby allowing for better targeted delivery with fewer off-target effects. In cases such as Friedreich's ataxia (FRDA), which is characterized by mitochondrial dysfunction, drug conjugated NPs can be combined with gene therapy for enhancing the delivery of genetic material to the target cells. Moreover, when NSC (neural stem cell) differentiation-based therapies are combined with NPs, they can help address the complexities of NDs by facilitating tissue regeneration and repair (70). Recent breakthroughs in NSC-targeting system are a convincing prospect for the realization of safer and more efficacious neuro-dystrophy therapies.

Nanomedicine is already proving valuable in treating tumors like glioblastoma, and emerging applications of nanoimmunotherapy are ushering in new opportunities to apply immunotherapy for neurodegenerative conditions (211). NPs can administer immunotherapies directly to specific immune cells, aiding and strengthening the body's natural immune response to neoplastic conditions or NDs. Nanomedicines can target a variety of immune-modulating agents, including antibodies, cytotoxic drugs, and vaccine antigens within the tumor microenvironment (TME) (212). Additionally, NPs can also be engineered to be biodegradable and stimuli-responsive, with the ability to control the release of their cargo in response to internal signals (e.g., pH, temperature) (213). These technologies can potentially enhance the effectiveness of immune responses and reduce adverse effects. Immunotherapies targeting specific pathological pathways in the brain have been developed for NDs like PD and AD. Nonetheless, hurdles persist, especially regarding the targeting of α-synuclein in PD, as the protein has multiple conformational states that pose a challenge to its targeting (214). Advances in the development of biocompatible nanoscale materials and the fine-tuning of immunotherapies will be crucial in overcoming these obstacles (215). Furthermore, the immune systems of older patients, particularly those with NDs, may be compromised, making it difficult to elicit an optimal immune response. As such, careful consideration must be given to the development of nanomedicine-based immunotherapies that can activate both innate and adaptive immunity (216).

Even though there has been a lot of progress in nanomedicine, the translation of NP-based therapies into clinical practice remains hindered by challenges related to their size, stability, and biodistribution. In order to improve clinical feasibility, the next generation of NP systems must have controlled biodistribution, reduced toxicity, and be administered less frequently (enhancing patient compliance). Designing nanoparticles to target a broad range of mechanisms is difficult, as different illnesses attack the brain in different ways. Moreover, animal models, such as rodents, often do not accurately represent human neural systems. Thus, a lot of onus is on refining animal models and improving patient stratification to consider individual genetic and phenotypic variations in neurological diseases for the success of NP-based systems in clinical practice. The next generation of nanomedicines should be designed to be safer, more selective, and more therapeutic, targeting the real degenerative triggers with reduced adverse effects (217).

## 10 Conclusion

Nanomedicine is a promising strategy for CNS disorders, and the synergy between nanomaterials at the nanoscale and gene therapy approaches to neural stem cell (NSCs) differentiation and immune modulation appears to be highly attractive. Conversely, overcoming the blood-brain barrier, nanoparticle toxicity, and patient variability still remain as some of the obstacles that require further research. Machine learning, AI, advanced models, and many other technologies are accelerating nanoparticle therapeutic tools on an even faster path to clinical translation. As nanotechnology develops, more specialized and accurate treatments for neurological disorders will be devised, providing hope for neurological illness patients.
